# Immune checkpoint inhibitor-induced neurotoxicity is not associated with seroprevalence of neurotropic infections

**DOI:** 10.1007/s00262-023-03498-0

**Published:** 2023-08-22

**Authors:** C. Schmitt, E. P. Hoefsmit, T. Fangmeier, N. Kramer, C. Kabakci, J. Vera González, J. M. Versluis, A. Compter, T. Harrer, H. Mijočević, S. Schubert, T. Hundsberger, A. M. Menzies, R. A. Scolyer, G. V. Long, L. E. French, C. U. Blank, L. M. Heinzerling

**Affiliations:** 1 Department of Dermatology and Allergy, University Hospital, LMU Munich, Munich, Germany; 2 Department of Molecular Oncology and Immunology, Netherlands Cancer Institute, Amsterdam, The Netherlands; 3 Department of Dermatology, Uniklinikum Erlangen and Friedrich–Alexander-Universität (FAU) Erlangen–Nürnberg, Erlangen, Germany; 4 Department of Medical Oncology, Netherlands Cancer Institute, Amsterdam, The Netherlands; 5 Department of Neuro-Oncology, Netherlands Cancer Institute, Amsterdam, The Netherlands; 6 Department of Internal Medicine 3, Infectious Diseases and Immunodeficiency Section, Universitätsklinikum Erlangen, Friedrich–Alexander-Universität Erlangen–Nürnberg, Erlangen, Germany; 7 Deutsches Zentrum Für Immuntherapie (DZI), Friedrich–Alexander-Universität Erlangen–Nürnberg (FAU) and Universitätsklinikum Erlangen, Erlangen, Germany; 8 Max Von Pettenkofer Institute of Hygiene and Medical Microbiology, Faculty of Medicine, LMU Munich, Munich, Germany; 9 Departments of Neurology and Medical Oncology/Haematology, Cantonal Hospital, St. Gallen, Switzerland; 10 Melanoma Institute of Australia, The University of Sydney, Sydney, NSW Australia; 11 Faculty of Medicine and Health, The University of Sydney, Sydney, NSW Australia; 12 Department of Medical Oncology, Royal North Shore and Mater Hospitals, Sydney, NSW Australia; 13 Charles Perkins Centre, The University of Sydney, Sydney, NSW Australia; 14 Tissue Pathology and Diagnostic Oncology, Royal Prince Alfred Hospital and NSW Health Pathology, Sydney, NSW Australia; 15 Dr. Philip Frost, Department of Dermatology and Cutaneous Surgery, University of Miami Miller School of Medicine, Miami, FL USA; 16 Department of Medical Oncology, Leiden University Medical Center, Leiden, The Netherlands

**Keywords:** Checkpoint-inhibitor, Side effect, Serology, Melanoma

## Abstract

**Background:**

Immune checkpoint inhibitors (ICI) substantially improve outcome for patients with cancer. However, the majority of patients develops immune-related adverse events (irAEs), which can be persistent and significantly reduce quality of life. Neurological irAEs occur in 1–5% of patients and can induce severe, permanent sequelae or even be fatal. In order to improve the diagnosis and treatment of neurological irAEs and to better understand their pathogenesis, we assessed whether previous neurotropic infections are associated with neurological irAEs.

**Methods:**

Neurotropic infections that might predispose to ICI-induced neurological irAEs were analyzed in 61 melanoma patients from 3 countries, the Netherlands, Australia and Germany, including 24 patients with neurotoxicity and 37 control patients. In total, 14 viral, 6 bacterial, and 1 protozoal infections previously reported to trigger neurological pathologies were assessed using routine serology testing. The Dutch and Australian cohorts (NL) included pre-treatment plasma samples of patients treated with neoadjuvant ICI therapy (OpACIN-neo and PRADO trials; NCT02977052). In the Dutch/Australian cohort a total of 11 patients with neurological irAEs were compared to 27 control patients (patients without neurological irAEs). The German cohort (LMU) consisted of serum samples of 13 patients with neurological irAE and 10 control patients without any documented irAE under ICI therapy.

**Results:**

The association of neurological irAEs with 21 possible preceding infections was assessed by measuring specific antibodies against investigated agents. The seroprevalence of all the tested viral (cytomegalovirus, Epstein-Barr-Virus, varicella-zoster virus, measles, rubella, influenza A and B, human herpes virus 6 and 7, herpes simplex virus 1 and 2, parvovirus B19, hepatitis A and E and human T-lymphotropic virus type 1 and 2), bacterial (*Borrelia burgdorferi *sensu lato*, Campylobacter jejuni, Mycoplasma pneumoniae, Coxiella burnetti, Helicobacter pylori, Yersinia enterocolitica* and *Y. pseudotuberculosis*) and protozoal (*Toxoplasma gondii*) infections was similar for patients who developed neurological irAEs as compared to control patients. Thus, the analysis provided no evidence for an association of described agents tested for seroprevalence with ICI induced neurotoxicity.

**Conclusion:**

Previous viral, bacterial and protozoal neurotropic infections appear not to be associated with the development of neurological irAEs in melanoma patients who underwent therapy with ICI across 3 countries. Further efforts are needed to unravel the factors underlying neurological irAEs in order to identify risk factors for these toxicities, especially with the increasing use of ICI in earlier stage disease.

## Introduction

Immune checkpoint inhibitors (ICI) have proven efficacy across multiple malignancies, significantly improving outcomes for many cancer patients [1]. As immune checkpoints are involved in self-tolerance and limiting of autoimmunity, ICI therapy can induce immune-related adverse events (irAEs) mimicking autoimmune disease. Severe irAEs, classified as grade 3–5 according to the Common Terminology Criteria for Adverse events (CTCAE), can be observed in 20–59% of patients [2]. Although any organ system can be affected, neurological toxicities are highly relevant irAEs due to their morbidity and mortality as well as permanent consequences. Neurological irAEs are observed in 1–5% [2] of patients, and can involve the central (CNS) as well as the peripheral nervous system (PNS) and the neuromuscular junction [3–7].

Reported neurological irAEs include immune neuropathies, like de novo manifestations or exacerbations of pre-existing immune-mediated neurological diseases such as Guillain–Barré like syndrome (GBS) [5], demyelinating polyneuropathy [4], enteric neuropathy [5], myasthenia gravis (MG) [5, 6, 8], multiple sclerosis (MS), or (vasculitic) neuropathies [9], but also posterior reversible encephalopathy syndrome, aseptic meningitis, (transverse) myelitis, and immune encephalitis [4, 5, 10]. Furthermore, cranial nerve involvement of optical and vestibulocochlear nerve can lead to vision impairment, hearing loss and vertigo [11]. Persistent irAEs after cessation of ICI therapy have been shown to severely reduce quality of life for cancer survivors [12], thus it would be of utmost importance to detect patients with a high risk of therapy-induced sequelae before starting ICI [13]. Additionally, since ICI are applied in earlier tumor stages, there is a dire need to carefully weigh risks and benefits [14]. So far the efforts to identify predictive markers for severe or life-threatening irAEs have been less fruitful than anticipated [15]. For neurological side effects this attempt is especially pressing since they are associated with a mortality rate of up to 21% [4], often require intensive care with e.g. in 27–37% of cases of ICI-induced encephalitis [10], and result in permanent sequelae in 11% of cases [4].

ICI-induced neurological irAEs behave differently from their autoimmune counterparts with a lower frequency of autoantibodies [7, 16], and different response to therapy. However, both conditions can be triggered by distinct organ-specific antigens. Peripheral neuropathies can be caused by immunological cross-reactivity in which an immune response to an environmental agent (e.g. infectious agent or vaccine) targets self-antigens from the nervous system [17, 18]. Even though the research field is hampered by latency, unknown interactions with the microbiota, and other environmental factors, molecular mimicry can play a role in autoimmune reactions [19]. GBS has been linked to autoantibody production after *Campylobacter jejuni* infection via molecular mimicry [17, 18]. In analogy to these autoimmune diseases, preceding infections could predispose to ICI-induced toxicity, especially neurological irAEs potentially associated with infectious agents reported to be causative for autoimmune neurological diseases [4, 18, 19]. Activation or re-activation of such previous immune responses by ICI therapy might trigger neurological irAEs by molecular mimicry.

Therefore, we tested whether exposure to a neurotropic infection is associated with the development of an ICI induced neurological irAE. This study analyzed 61 cancer patients for serological evidence of 21 infectious agents and their possible association with the occurrence of neurological irAE following ICI.

## Methods

### Patient population

**Fig. 1:**
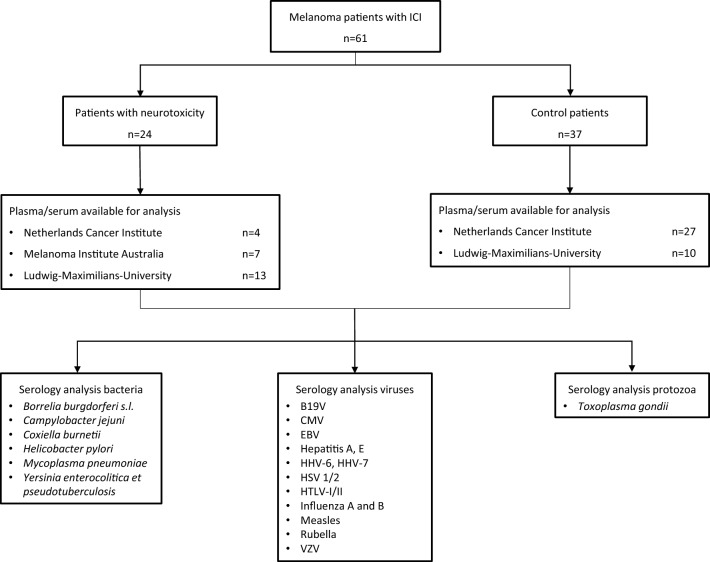
Study overview. Analysis of melanoma patients treated with immune checkpoint inhibitor (ICI) (n = 61). The samples of the Netherlands Cancer Institute (NKI) and Melanoma Institute Australia (MIA) were plasma samples (NL) and the samples of the Ludwig–Maximilians University (LMU) were serum samples. A total of 24 patients with neurological irAEs was compared to 37 patients without neurotoxicity. Patients mainly received ipilimumab plus nivolumab combination therapy (all NL, part of LMU), and the percentage receiving monotherapy and combination therapy was balanced (Table 2). Abbreviations: sensu lato (s.l.), parvovirus B19 (B19V), cytomegalovirus (CMV), Epstein-Barr virus (EBV), human herpesvirus (HHV), herpes simplex virus (HSV), human T-lymphotropic virus (HTLV), varicella-zoster virus (VZV)

Plasma and serum samples of patients treated with ICI were identified from three cancer centers (Netherlands Cancer Institute (NKI), Melanoma Institute Australia (MIA) and Ludwig–Maximilians-University (LMU), Germany) (Fig. 1). This cohort included pre-treatment plasma samples of patients of the OpACIN-neo (n = 32) and PRADO trials (n = 6; NCT02977052) and serum samples of the LMU (n = 23).

In the OpACIN-neo and PRADO trials (NCT02977052), summarized as Dutch cohort (NL), melanoma patients were treated with different doses of neoadjuvant ipilimumab and nivolumab [20–22]. At baseline all 38 patients were stage III according to AJCC 2017. Out of this cohort, 11 patients developed neurological irAEs, ranging from grade 1 to 5 CTCAE (Table 1). Patients treated at the NKI within the OpACIN-neo trial who did not experience neurological irAEs were selected as control cohort (n = 27). The NL control cohort included patients who experienced other types of irAEs such as gastrointestinal irAEs, irArthritis, irHepatitis or irDermatitis, of which 10 demonstrated CTCAE grade 3 or 4 irAEs.

**Table 1:** Methods of investigated infectiological agents

Infectiological agents	Method	Details [unit]	Cut-off range
*Virological agents*			
CMV IgG	CLIA	Quantitative [U/ml]	12–14
EBV-EA IgG	CLIA	Quantitative [U/ml]	10–40
EBV-EBNA IgG			20–40
EBV-VCA IgG			20
Hepatitis A IgG	CMIA	Semiquantitative	0.8–1.1
Hepatitis E IgG	ELISA and CLIA	Semiquantitative Quantitative [AU/ml]	0.8–1.1
HHV-6 IgG	ELISA	Semiquantitative	0.9–1.1
HHV-7 IgG	IFT	Semiquantitative	1:8
HSV 1/2 IgG	CMIA	Semiquantitative	0.9–1.1
HTLV-I/II IgG	CMIA	Semiquantitative	1
Influenza A IgG	ELISA	Semiquantitative	0.8–1.1
Influenza B IgG	ELISA	Semiquantitative	0.8–1.1
Measles IgG	CLIA	Quantitative [AU/ml]	13.5–16.5
B19V IgG	CLIA	Semiquantitative	0.9–1.1
Rubella IgG	CLIA	Quantitative [IU/ml]	7–10
VZV IgG	CLIA	Quantitative [mIU/ml]	50–100
*Bacteriological and protozoal agents*			
Coxiella burnetii IgG	CLIA	Semiquantitative	0.9–1.1
Coxiella burnetii IgM			0.9–1.1
Borrelia burgdorferi s.l. IgG	ELISA	Quantitative [AU/ml]	16–22
Borrelia burgdorferi s.l. IgM			16–22
Campylobacter jejuni IgG	ELISA	Quantitative [AU/ml]	16–22
Campylobacter jejuni IgA		Semiquantitative	0.8–1.1
Helicobacter pylori IgG	ELISA	Semiquantitative	0.8–1.1
Helicobacter pylori IgA			0.8–1.1
Mycoplasma pneumoniae IgG	ELISA	Quantitative [AU/ml]	16–22
Mycoplasma pneumoniae IgA		Semiquantitative	0.8–1.1
Mycoplasma pneumoniae IgM		Semiquantitative	0.8–1.1
Toxoplasma gondii IgG	ELISA	Quantitative [IU/ml]	8–11
Toxoplasma gondii IgM		Semiquantitative	0.8–1.1
Yersinia p.e. IgG	Immunoblot	Qualitative	–
Yersinia p.e. IgA			
Yersinia p.e. IgM			

Virological and bacteriological/protozoal agents are given in alphabetical order. Virological agents: Immunoglobulin G (IgG) antibodies measured using chemiluminescent immunoassay (CLIA) are indicated in arbitrary units per milliliter [AU/ml] as well as international units per milliliter [IU/ml] and units per milliliter [U/ml]. HHV-7 was detected by indirect immunofluorescence technique (IFT), classified as negative if the titer was below 1:8.Other methods: enzyme-linked immunosorbent assay (ELISA) and chemiluminescence microparticle immunoassay (CMIA). Bacteriological and protozoal agents: Immunoglobulins A, G, M (IgA, IgG, IgM) were semiquantitatively and quantitatively measured using CLIA and ELISA. Immunoglobulins against Yersinia enterocolitica and pseudotuberculosis (Yersinia p.e.) were detected by immunoblot, in which case they were only either detected or not detected (positive/negative). Anti-phase-II-Coxiella burnetii immunoglobulins were measured, testing for phase-II-antigen, which is more likely in an acute than in a chronic infection. Cut-off range: values in the displayed range were classified as inconclusive, seroprevalence below and above cut-off points was considered as negative and positive. If only one cut-off point is displayed, values below were classified as negative

The LMU cohort consisted of 23 melanoma patients receiving ipilimumab plus nivolumab or ICI monotherapy (nivolumab, pembrolizumab or ipilimumab) including longitudinal blood samples (Table 2). Out of these, 13 patients developed neurological irAEs and 10 served as control patients. 30 samples were collected for the irAE group, including 6 baseline (*bl*), 12 under ICI therapy (*cxp*) and 12 at the time of the adverse event (*ae*). Either *cxp* or *ae* or both were at least obtained from every irAE patient. The LMU control cohort included 10 patients who did not develop any kind of irAE, with one sample per patient, split into 1 at *bl* and 9 at *cxp* time point. Due to differing serum volumes for each timepoint, some parameters could not be measured in all samples.

**Table 2:** Baseline characteristics of study cohort treated with ICI

Baseline characteristics	Control cohort		Neurological irAE		Total cohort	
	NL	LMU	NL	LMU	irAE	Control
n	27	10	11	13	24	37
Age (years)	53 (27–78)	69 (31–80)	58 (24–70)	58 (42–74)	58 (24–74)	57 (27–80)
Gender: female—male: n (%)	13—14 (48%—52%)	2—8 (20%—80%)	8—3 (73%—27%)	6—7 (46%—54%)	14—10 (58%—42%)	15—22 (41%—59%)
*Primary tumor stage*						
T1a/b	8 (30%)	0 (0%)	4 (36%)	2 (15%)	6 (25%)	8 (22%)
T2a/b	4 (15%)	3 (30%)	0 (0%)	1 (8%)	1 (4%)	7 (19%)
T3a/b	5 (19%)	2 (20%)	2 (18%)	1 (8%)	3 (12%)	7 (19%)
T4a/b	2 (7%)	1 (10%)	2 (18%)	4 (31%)	6 (25%)	3 (8%)
Tx	8 (30%)	0 (0%)	1 (9%)	2 (15%)	3 (12%)	8 (22%)
Unknown primary	0 (0%)	4 (40%)	2 (18%)	3 (23%)	5 (21%)	4 (11%)
*Primary AJCC stage*						
I	0 (0%)	1 (10%)	0 (0%)	1 (8%)	1 (4%)	1 (3%)
II	0 (0%)	3 (30%)	0 (0%)	1 (8%)	1 (4%)	3 (8%)
III B/C	27 (100%)	2 (20%)	11 (100%)	1 (8%)	12 (50%)	29 (78%)
IV	0 (0%)	4 (40%)	0 (0%)	10 (77%)	10 (42%)	4 (11%)
*Therapy regimen ICI*						
Pembrolizumab/Nivolumab	0 (0%)	6 (60%)	0 (0%)	2 (15%)	2 (8%)	6 (16%)
Ipilimumab	0 (0%)	0 (0%)	0 (0%)	3 (23%)	3 (12%)	0 (0%)
Ipilimumab + Nivolumab	27 (100%)	4 (40%)	11 (100%)	8 (62%)	19 (79%)	31 (84%)
*Prior therapy*						
Chemotherapy	0 (0%)	0 (0%)	0 (0%)	5 (38%)	5 (21%)	0 (0%)
Radiotherapy	0 (0%)	1 (10%)	0 (0%)	0 (0%)	0 (0%)	1 (3%)
IFN-α	0 (0%)	1 (10%)	0 (0%)	0 (0%)	0 (0%)	1 (3%)
BRAF-/MEK-inhibitor	0 (0%)	0 (0%)	0 (0%)	5 (38%)	5 (21%)	0 (0%)

Percentages may not sum up to 100 due to rounding. Age: median displayed with range in brackets. NL cohort included OpACIN-neo and PRADO trial patients from the Netherlands and Australia, LMU included German patients from the MelAutim study. Primary AJCC 2017 stage and prior therapy before initiation of ICI displayed

Clinical data was obtained from eCRFs and electronic patients files. Ethical consent was obtained within the OpACIN-neo and PRADO studies (NL) and the MelAutim study (LMU) (No. 20-1122) from the respective institutional review boards. Patients gave written informed consent before inclusion. Adverse events were graded using CTCAE version 5.0 [23].

### Infectiological analyses

A total of 14 viral, 6 bacteriological and one protozoal agents was investigated for seroprevalence. Due to different available sample volumes for each patient, a priorization was established, taking different variables into account such as needed amount of volume and interest in the parameter. For this reason, the numbers of investigated samples slightly vary for single parameters. Selected infections were based on possible associations with neurologic symptoms. Previously described associations with ICI were also taken into account, such as human T-lymphotropic virus (HTLV) [24], *Helicobacter pylori* [25], *Toxoplasma gondii* [26], influenza [27], cytomegalovirus (CMV) [28], *Campylobacter jejuni* [29] or Epstein–Barr virus (EBV) [30] to finally come up with the curated list of interest of potentially neurotropic infectious agents.

#### Viral serology

Seroprevalence was assessed using chemiluminescent immunoassay (CLIA), chemiluminescence microparticle immunoassay (CMIA), enzyme-linked immunosorbent assay (ELISA) and indirect immunofluorescence technique (IFT). These assays provide quantitative or semiquantitative readouts, respectively (Table 1).

Antibodies against CMV, EBV, measles, parvovirus B19 (B19V), rubella and varicella-zoster virus (VZV) were measured using CLIA. For EBV, three different parameters were measured including antibodies against early antigen (EA), Epstein-Barr nuclear antigen (EBNA) and viral capsid antigen (VCA), respectively, each related to a different stage of this viral infection. Specific antibodies against hepatitis A, herpes simplex virus (HSV 1/2), as well as HTLV type I/II were detected using CMIA, whereas antibodies against hepatitis E, human herpesvirus 6 (HHV-6), influenza A and B were quantified using ELISA. Measurement of hepatitis E-antibodies was first performed using ELISA but was switched to CLIA. Lastly, IFT was applied to measure antibodies against human herpesvirus 7 (HHV-7).

#### Bacterial and protozoal serology

For investigated bacterial and protozoal agents, quantitative, semiquantitative and qualitative methods were applied, using ELISA, CLIA and immunoblotting (Table 1). In comparison to the viral serology, where only IgG's were quantified, bacteriological agents were additionally measured as IgM and/ or IgA. Antibodies against phase-II-antigens of *Coxiella burnetii* were investigated using CLIA, antibodies against *Yersinia enterocolitica* and *Y. pseudotuberculosis* (summarized as *Yersinia p.e.*) by using immunoblotting, whereas the other parameters were measured by ELISA (*Borrelia burgdorferi *sensu lato* (s.l.), Campylobacter jejuni, Helicobacter pylori, Mycoplasma pneumoniae* and the protozoa *Toxoplasma gondii*).

### Statistical analysis

The NL cohort and the LMU cohort were analyzed separately as well as combined, considering possible differences such as center effects. Analyses were performed using GraphPad Prism 9.3.1 (GraphPad Software, Inc. San Diego, California, USA), graphs were generated using GraphPad Prism 9.3.1 and Microsoft PowerPoint 2016. Comparisons were made using Mann–Whitney-U-tests and/or Kruskal–Wallis-tests with a significance level of *p* ≤ 0.05.

For all measured parameters, the neurotoxicity group was compared to the control group. Additionally, since irHypophysitis has been classified as neurological or endocrine irAE, cases of the neurotoxicity group with irHypophysitis were analyzed separately in comparison to the control group to unravel possible effects of this type of irAE on the results. The quantitative parameters were visualized as scatter dot plots, displayed with median and 95% confidence intervals (CI). Semiquantitative and qualitative parameters were visualized as stacked bar graphs.

## Results

### Patient population

Melanoma patients receiving ICI therapy were included for seroprevalence analyses of 21 bacterial, viral and protozoal agents described in the context of neurological pathologies (Table 1). Samples were collected from three different countries (Netherlands, Australia, Germany) with plasma samples before initiation of ICI therapy in the trial patients (NL) or serum samples of at least one of three possible time points in patients treated in Germany (LMU).

In the NL cohort 11 patients reported neurological irAEs (Table 2), such as meningitis, encephalitis, transverse myelitis, ataxia and different neuropathies, including a bilateral ulnar neuropathy, autonomic neuropathy (gastroparesis), bilateral Bell's palsy, and polyradiculoneuropathy (Table 3). The control cohort consisted of 27 patients without neurological irAEs. All included NL patients received neoadjuvant ipilimumab plus nivolumab without any prior systemic therapy or radiotherapy.

**Table 3:** Neurological adverse events in investigated cohorts of melanoma patients under ICI treatment

Neurological irAE	NL				LMU				Total cohort				
n	11 patients, 12 diagnoses				13 patients, 14 diagnoses				24 patients, 26 diagnoses				
CTCAE grade	1–2	3	4	5	1–2	3	4–5	Unknown	1–2	3	4	5	Unknown
n (%)	4 (33)	6 (50)	1 (8.3)	1 (8.3)	5 (36)	8 (57)	0 (0)	1 (7)	9 (35)	14 (54)	1 (4)	1 (4)	1 (4)
Ataxia		1								1			
Bell’s palsy		1								1			
Encephalitis				1		1				1		1	
Gastroparesis	1								1				
Hypophysitis					5	4		1	5	4			1
Meningitis	1	2							1	2			
Myelitis						1				1			
Neuritis*						1				1			
Neuropathy	1	1	1						1	1	1		
Radiculitis	1								1				
Transverse myelitis-like syndrome		1								1			
Vestibulopathy						1				1			

Numbers of cases displayed (percentages in brackets). Alphabetical order of neurological irAEs. In our case the specific differentiation of ataxia as a symptom for i.e. polyneuropathy was not possible. Percentages may not sum up to 100 due to rounding. *Neuritis case could potentially have polyneuropathy, whereas neuritis was more probable. One patient in each cohort appears twice because of two leading diagnoses of one patient. Common Terminology Criteria for Adverse events (CTCAE v 5.0) used for grading of irAEs

LMU patients received ICI combination therapy with ipilimumab plus nivolumab (62% of irAE group, 40% of controls) or ICI monotherapy with pembrolizumab, nivolumab or ipilimumab (38% or irAE, 60% of control group) (Table 2).

The LMU cohort contained 13 patients with neurological irAEs such as neuritis, myelitis, encephalitis or vestibulopathy (Table 3). Of these, 10 patients had received a prior systemic therapy with chemotherapy (38%) or BRAF-/MEK-inhibitors before initiation of ICI (38%). The LMU control group included 10 patients who received ICI therapy but did not develop any kind of irAE (Table 2). A part of these had received radiotherapy (10%) or interferon (IFN)-alpha therapy (10%) before ICI. For further details of patients’ characteristics, see Tables 2 and 3.

### Virological results

#### Quantitative parameters

**Fig. 2:**
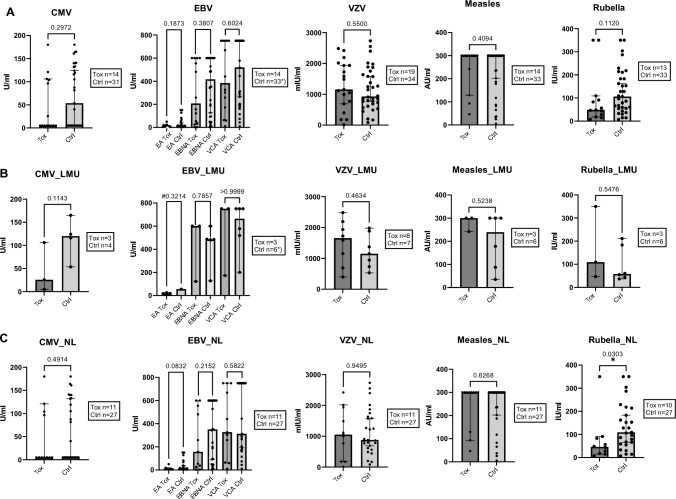
No association of quantitative agents for viral infections with neurological irAEs compared to controls without neurological irAE. Melanoma patients treated with ICI with (Tox) and without (Ctrl) neurological irAEs. Values of IgG antibodies against each virus measured. Combined (**A**) and separate analyses of LMU (**B**) and NL (**C**) cohorts. EBV *): differing n of Ctrl, (A) Ctrl n = 28 for EA, n = 32 for EBNA, n = 33 for VCA; (B) EBNA Ctrl n = 5. (C) Rubella virus antibodies statistically significant in NL cohort, Ctrl > Tox, p = 0.0303. (B) EBV EA #: no value comparison possible (Tox n = 3, Ctrl n = 1), p of pos/neg comparison displayed with Tox n = 3 and Ctrl n = 6. (A) CMV: In the Tox group 8/14 (57%) (1 from LMU (B) and 7 from NL (C)) were classified negative, in the Ctrl group this was the case for 14/31 (45%) (all NL (C)) cases. P values displayed. Boxes show the numbers of measured samples for each agent. Control group consisted of patients without neurological irAEs (NL) and of patients without any ICI-induced irAE (LMU). Mann–Whitney-U tests. Median with 95% CI displayed. For reference cut-off points see Table 1. Serum (LMU, B) and plasma (NL, C) analyses

Seroprevalence of viral pathogens described to potentially induce neurological pathologies were investigated for the cohorts of melanoma patients under ICI (Table 1). Samples were analyzed for the quantitative measurement of antibodies against cytomegalovirus (CMV), Epstein-Barr-virus (EBV), varicella-zoster virus (VZV), measles virus and rubella virus (Fig. 2).

For EBV, antibodies against early antigen (EA), Epstein-Barr-nuclear antigen (EBNA) and viral capsid antigen (VCA) were measured. For described quantitative viral agents, patients who developed neurological irAEs after initiation of ICI therapy (Tox) did not have significantly higher specific IgG levels compared to the control cohorts (Ctrl), considering combined (Fig. 2A) and separate analyses of the LMU (Fig. 2B) and NL cohort (Fig. 2C). Investigating the NL cohorts separately, significantly higher specific IgG levels of antibodies against rubella virus were found in the control group compared to patients that developed neurological irAEs (Fig. 2C).

In addition, irHypophysitis was analyzed separately for VZV (LMU), in comparison to other neurological irAEs and controls (Kruskal–Wallis-test). There was no significant difference for VZV (*p* = 0.4407). No irHypophysitis only case was included in other quantitative viral measurements due to priorization and available amounts of volume.

#### Semiquantitative parameters

**Fig. 3:**
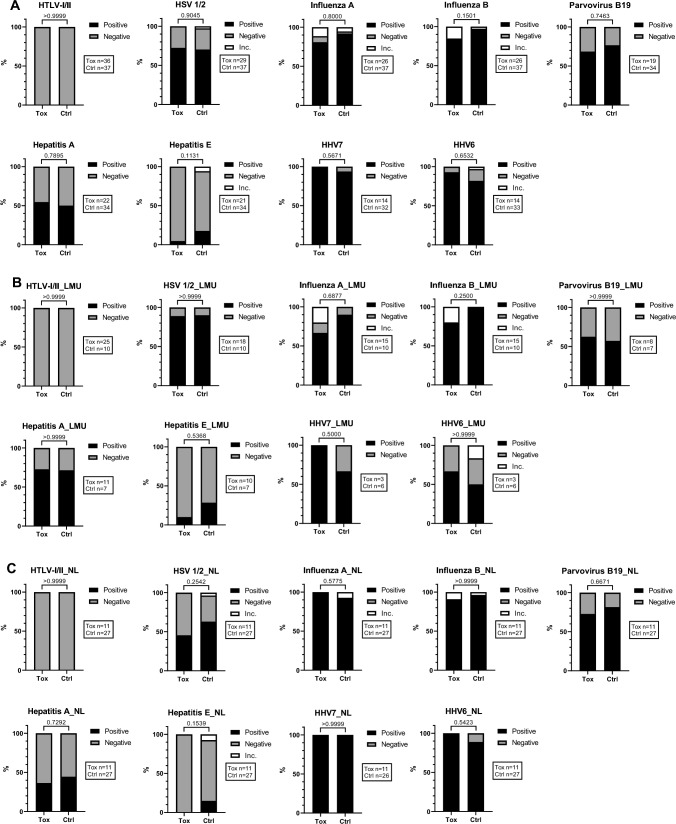
Semiquantitative virological agents are not associated with neurological irAEs. Melanoma patients treated with ICI with (Tox) and without (Ctrl) neurological irAEs. Combined (**A**) and separate analyses of LMU (**B**) and NL (**C**) cohorts. Evaluation of positive, negative or inconclusive (Inc.) values for each semiquantitative parameter. P values displayed. Boxes show the number of measured samples for each agent. Control group consisted of patients without neurological irAEs (NL) and of patients without any ICI-induced irAEs (LMU). Mann–Whitney-U tests. For reference cut-off points see Table 1. Serum (LMU) and plasma (NL) analyses compared

In addition to quantitative parameters, we determined antibodies against virological parameters semiquantitatively, against influenza A and B, HHV6 and HHV7, HSV 1/2, B19V, hepatitis A and E and HLTV-I/II (Fig. 3A–C).

The majority of patients tested positive for influenza A and B viruses, HSV 1/2, HHV6, HHV7 and B19V, whereas most tested negative for hepatitis E and all patients tested negative for HLTV-I/II.

There was no significant association between these semiquantitative virological agents and neurological irAEs when comparing to the control patients, in combined (Fig. 3A) as well as separate analyses of the cohorts (Fig. 3B, C).

As described for the quantitative viral parameters, no significant difference was observed comparing irHypophysitis cases to other neurological irAEs and to the controls (LMU), conducted for the semiquantitative virological parameters HSV 1/2 (*p* = 0.4066), influenza virus A (*p* = 0.4204) and B (*p* = 0.1699), B19V (*p* > 0.9999) as well as for hepatitis virus A (*p* = 0.2386) and E (*p* = 0.7838). Also for other semiquantitative virological parameters, no irHypophysitis case was measured (HHV6 and 7) or all investigated samples were negative (HTLV-I/II).

The data implicate that there is no association between the tested quantitatively and semiquantitatively assessed virus-specific antibodies (Table 1) and the development of neurological irAEs under ICI therapy in our cohorts (Figs. 2 and 3).

### Bacteriological and protozoan results

#### Quantitative parameters

**Fig. 4:**
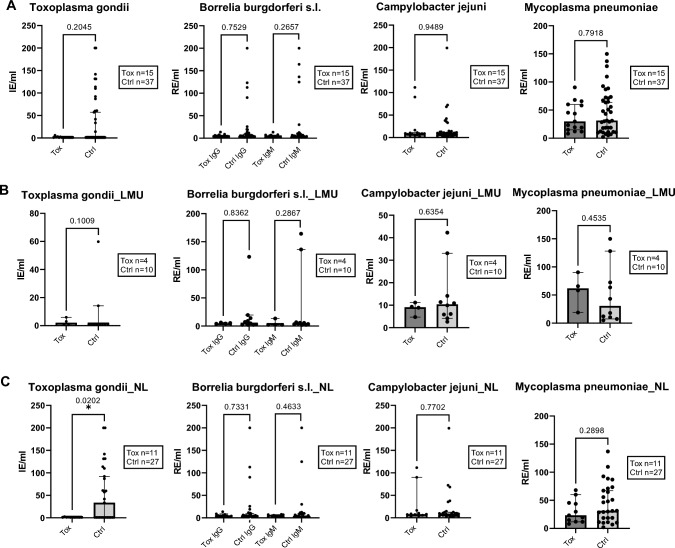
Bacteriological and protozoal quantitative agents are not associated with neurological irAEs. Values of antibodies against each bacterial/protozoal parameter. (**A**) Combined analysis of NL and LMU cohorts. Separate analyses of LMU (**B**) and NL (**C**) cohorts. *Toxoplasma gondii* EIA IgG, statistically higher values of Ctrl compared to Tox in NL cohort only. Borrelia EIA IgG and IgM. *Campylobacter jejuni* EIA IgG. *Mycoplasma pneumoniae* EIA IgG. P values displayed. Boxes show the numbers of measured samples for each agent. Comparison of patients with neurological irAE (Tox) under ICI vs. controls (Ctrl). Control group consisted of patients without neurological irAEs (NL) and of patients without any ICI induced irAEs (LMU). Mann–Whitney-U tests. Median with 95% CI displayed. For reference cut-off points see Table 1. Plasma (NL) and serum (LMU) analyses. Antibody concentrations per mililiter (ml): Internationale Einheit = international unit (IE), relative Einheiten = relative units (RE)

Melanoma patients treated with ICI were analyzed for neurotropic infections with several bacterial and protozoal pathogens (Table 1). Antibodies against *Toxoplasma gondii* (IgG), *Borrelia burgdorferi* s.l. (IgG and IgM), *Campylobacter jejuni* (IgG) and *Mycoplasma pneumoniae* (IgG) were determined by quantitative assays (Fig. 4). Samples were either pre-treatment plasma samples (NL) or serum samples (LMU), analyzed combined (Fig. 4A) and separately (Fig. 4B, C).

**Fig. 5:**
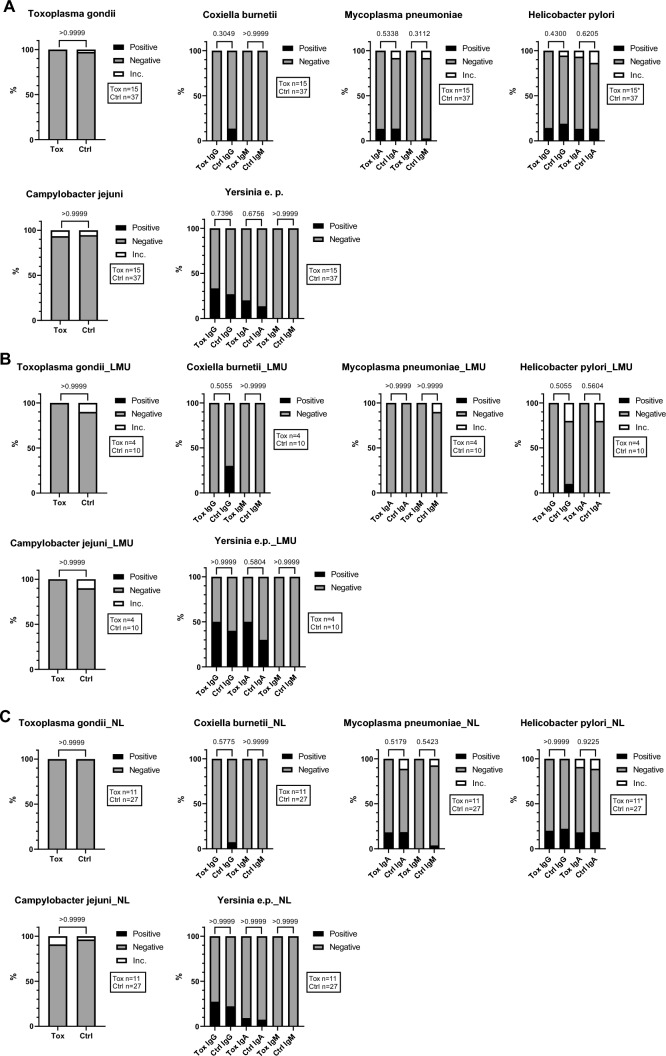
Bacteriological and protozoal semiquantitative and qualitative agents showed no association with neurological irAE. Melanoma patients with neurological irAE under ICI (Tox) versus control (Ctrl). Semiquantitative examination of antibodies against each bacterial/protozoal parameter. Combined (**A**) and separate analysis of NL (**C**) and LMU (**B**) cohorts. Toxoplasma gondii EIA IgM. Helicobacter pylori IgG and IgA, (A)*: for IgG Tox n = 14, (C) *: for IgG Tox n = 10. Campylobacter jejuni IgA. P values displayed. Boxes show the numbers of measured samples for each agent. Comparison of patients with neurological irAE (Tox) under ICI vs. controls (Ctrl). Control group included patients without neurological ICI induced irAEs (NL) and patients without any ICI induced irAEs (LMU). Mann–Whitney-U tests. For reference cut-off points see Table 1. Plasma (NL) and serum (LMU) analyses

Patients developing neurological irAEs under ICI therapy (Tox) did not have significantly higher specific IgG levels compared to the control patients without neurological irAEs (Ctrl). In the NL cohort alone, significantly higher IgG levels against *Toxoplasma gondii* were observed for control patients (*p* = 0.0202) (Fig. 4C), but all patients were determined to be IgM negative (Fig. 5C).

A separate analysis of irHypophysitis cases in comparison to other neurological irAEs and to controls was not conducted since only one case of isolated irHypophysitis was investigated regarding the quantitative bacteriological and protozoal parameters.

#### Semiquantitative parameters

Regarding semiquantitative and qualitative analyses of anti-bacterial antibodies and anti-Toxoplasmosis IgM antibodies, the majority of patients tested negative for *Campylobacter jejuni* IgA and for *Mycoplasma pneumoniae* IgA and IgM, in both the combined (Fig. 5A) and separate analysis of the cohorts (Fig. 5B, C). In addition, most patients were negative for antibodies against *Coxiella burnetii* (IgG and IgM), *Helicobacter pylori* (IgG and IgA) and *Yersinia e.p.* (IgG, IgA and IgM). No significant difference between patients with or without neurological irAEs was observed (Fig. 5). A separate analysis of irHypophysitis in comparison to other neurological irAEs and controls was not performed, since only one case was included in the semiquantitative measurement of anti-bacteriologal and anti-protozoal antibodies.

Together, this implicates that in our patient cohorts, there was no association between the development of neurological irAEs under ICI therapy and serological signs of previous or current neurotropic bacterial and protozoal infections with the tested pathogens (Table 1, Fig. 4, 5).

## Discussion

Treatment with ICI is increasingly common and irAEs constitute a considerable obstacle for safe administration, especially in the case of the life-threatening and irreversible neurological irAEs. Therefore, a deeper understanding of the aetiology and risk factors of neurological irAEs is of great importance. Immunological cross-reactivity of T-cells in response to infectious agents and host antigens from the nervous system could predispose patients to develop neurological irAEs under ICI due to molecular mimicry.

In this study, 61 melanoma patients under ICI therapy with (24 patients) and without (37 patients) neurological irAEs were tested for seroprevalence of 21 infections (14 viral, 6 bacterial and 1 protozoal), which have been previously described to trigger neurological pathologies potentially by molecular mimicry [17–19]. In two independent cohorts, there was no serological evidence for a higher incidence of preceding neurotropic infections in patients who developed neurological irAEs compared to patients without neurological irAEs. While this data suggests the absence of an association between neurotropic infections and development of neurological irAEs under ICI treatment, there are some limitations to our study. One limitation is that for some investigated parameters we observed a high serum prevalence in the control group as well as in the neurological irAE group, including influenza A and B, HSV 1/2 and parvovirus B19. This is in line with the general population, for which high incidences of these infections are known [31–33]. As only a minority of subjects infected with those pathogens may experience autoimmunity, we cannot rule out a participation of infections with these pathogens in the induction of neurological side effects either by molecular mimicry or by immune activation due to replication in the nervous system.

Since neurological irAEs are rare, and there is a large variety of types of neurological irAEs, it has previously been called for to join forces in the investigation of rare side effects to increase the number of patients per subtype [34], as it has been done for myocarditis [35]. Thus, maximizing collaboration efforts such as the side effect registry immune-oncology (www.serio-registry.org) [34] are needed especially in view of the high mortality rate reaching 21% for neurological irAEs. Additionally, irHypophysitis has been discussed controversially to be classified as a neurological or an endocrine irAE, whereas a strict classification is hardly possible [36, 37]. However, even when analysing irHypophysitis separately in comparison to the other investigated neurological irAEs or to the controls, no differences were observed. Nevertheless, by increasing the number of patients with a specific type of neurological irAEs, for example, irGBS [17], correlations with specific infections like *Campylobacter jejuni* infection or with specific HLA types might appear. This remains challenging due to the rarity of events. Taking the low incidence of neurological irAEs into consideration, this study analyzed a relatively high number of patients compared to previous studies [4, 5, 7, 10].

Few predictive markers for irAEs have shown an increased risk for irAEs in smokers, patients under 60 years of age, patients with a higher body mass index, and with severe lung, heart or kidney disease, whereas unspecific biomarkers such as cytokines or HLA genotypes have shown minor predictive evidence [15]. Increased inflammatory markers including c-reactive protein (CRP) and interleukin-6 are known to precede irAEs [38] and shifts in immune profiles occur early in the course of ICI treatment [13, 15]. For irThyreoiditis, baseline autoantibodies might be a risk factor [16]. *Helicobacter pylori* positivity has been demonstrated to be associated with decreased survival in ICI-treated patients [39], whereas only 4.3% of irGastritis cases under ICI are *Helicobacter pylori* and CMV positive [40]. This points out the relevance but also difficulty associated with the establishment of predictive markers for irAEs.

Importantly, our results were independent of investigated material, country and cohort with similar results for plasma and serum samples from the Netherlands (NL) and the German cohorts (LMU). We showed that there was no significant difference of the described viral, bacterial or protozoal pathogens with neurological irAEs across two different cohorts and three countries as well as no difference dependent on the tested material.

Significantly higher values of rubella IgG and *Toxoplasma gondii* IgG occurred in the NL control cohort compared to the NL neurological irAE cohort in contrast to the combined analysis (NL + LMU) or the LMU cohort alone. Retrospectively, it would have been of interest to query possible factors that may have an influence on the infectious parameters in the investigated cohorts. Since we were not able to survey these factors due to the already closed trials (OpACIN-neo and PRADO), we suppose a coincidentally occurring larger number of cat owners in the control group (NL) compared to the neurological irAE group could be causative for the observation regarding *Toxoplasma gondii* [41]. The higher levels of rubella IgG in the control group (NL) could be potentially explained by a higher number of participants with incomplete vaccination status or by a higher frequency of patients with contact to young children in this group, since infants of 0–4 years of age were observed to have the highest incidence in relation to age [42]. Therefore, we assume the results of these two parameters are of minor importance and occurred by coincidence. Also other tested agents might have been influenced by confounding factors and possibly appeared as false negative results. Therefore, we recommend the inclusion of a survey for unravelling the vaccination status and other potential influencing factors related to patients’ lives in future studies.

Even though no association was found in our cohorts to rule out mechanisms in the aetiology of neurological irAEs, antibodies or T cell clones directed against previously reported immunodominant epitopes have to be better characterized [18, 19]. Especially pre- and post-neurological symptom samples would be of interest for dynamics, particularly to investigate the development of serological IgM levels possibly indicative for a reactivation or a new infection. As an alternative to pre-existing infectious molecular mimicry, other possible underlying mechanisms could potentially trigger neurological irAEs. Autoreactive T and B cells to healthy tissue are thought to be a prime factor in the development of irAEs [19]. These autoreactive T cells could be pre-existing and perturbation of self-tolerance by ICI therapy could lead to reactivation, potentially due to genetic pre-disposition [43, 44]. In addition, a de novo autoreactive immune response could be generated through epitope spreading [19, 45]. These potential mechanisms are not mutually exclusive and could both contribute to (neurological) irAEs.

In conclusion, we did not find an association between the serologic prevalence for previous neurotropic infections in patients with melanoma and the development of neurological irAEs under ICI therapy. Given that neurological irAEs form a major obstacle for safely administering ICI in cancer patients, further efforts to unravel the underlying causes and predictive biomarkers of these toxicities are needed so as to identify patients at risk for irAEs induced by ICI treatment.
